# A pilot study: Use of the Adult Attention-Deficit/Hyperactivity Disorder Self-Report Scale in a South African patient population

**DOI:** 10.4102/sajpsychiatry.v25i0.1326

**Published:** 2019-05-22

**Authors:** Judith Regnart, Ilse Truter, Zukiswa Zingela, Anneke Meyer

**Affiliations:** 1 Department of Pharmacy, Nelson Mandela University, Port Elizabeth, South Africa; 2 Department of Psychiatry and Behavioural Sciences, Walter Sisulu University, Mthatha, South Africa; 3 Department of Psychology, Nelson Mandela University, Port Elizabeth, South Africa

**Keywords:** Attention-deficit/hyperactivity disorder, Substance use disorder, Adult ADHD self-report scale, Mental illness, Adult

## Abstract

**Background:**

Attention-deficit/hyperactivity disorder (ADHD) is a neurodevelopmental disorder which typically presents in childhood. This diagnosis may often be overlooked in adulthood, particularly in psychiatric populations. The Adult ADHD Self-Report Scale (ASRS) is an internationally used and reliable screener; however, studies investigating its use in African populations are limited.

**Aim:**

To investigate the application of the ASRS in a South African setting.

**Setting:**

A patient population in Port Elizabeth, South Africa, was identified as representing a developing or low- and middle-income country population.

**Methods:**

A convenience sample of acutely presenting psychiatric participants admitted for stabilisation was used. Fieldworkers administered the ASRS; collected information relating to demographics, differential diagnoses, substance use disorder (SUD) presence and substance consumption; and prescribed medication relating to current or historical treatment of ADHD.

**Results:**

The study sample included 30 participants, with black people representing the majority of participants. Adult ADHD Self-Report Scale completion revealed the rate of ADHD within the study population to be 43.3%, a contrast to the initially presumed prevalence of 6.7% which was based on reported methylphenidate therapy. A difference in SUD prevalence was identified between subjects screening positively and negatively for ADHD with a greater tendency towards SUDs seen for ASRS-positive individuals. Significant differences were identified in relation to cannabis- and polysubstance use for ASRS-positive individuals.

**Conclusion:**

Despite limitations related to the sample used and challenges in ASRS administration, investigation findings support recommendations for ADHD screening inclusion in acute inpatient settings in South Africa and ASRS translation into indigenous African languages.

## Introduction

Attention-deficit/hyperactivity disorder (ADHD) is defined as a neurodevelopmental disorder which is characterised by inattention, hyperactivity, impulsiveness and poor organisation.^1,2,3^ ADHD is fundamentally a cognitive disorder wherein there is a developmental impairment of executive functions (EFs) – complex cognitive processes that form the controlling system of the brain.^4,5,6,7^ According to Barkley,^8^ emotion is an important element to include when conceptualising ADHD. Emotions are not necessarily more intensely felt in persons with ADHD but are more quickly displayed and thus seem more marked. They are less moderated by executive self-regulation.

Commonly conceptualised as a childhood condition which is ‘outgrown’ during adolescence, ADHD has been described in adult populations for several decades, but its persistence in adulthood was only officially recognised in the fifth edition of the *Diagnostic and Statistical Manual of Mental Disorders (DSM-5)*.^1,9,10^ A key factor in its diagnosis is the degree to which the core symptoms of hyperactivity, impulsiveness and/or inattention result in functional impairment in different environments and domains.^1,3^ In adolescence and adulthood, hyperactive behaviours tend to decline, but impulsiveness- and/or inattention-related impairment(s) can impact adulthood contextual domains such as household management and work performance.^2,3,11,12^

The recognition of adult ADHD in psychiatric populations is often underreported. The diagnosis may be obscured by high rates of psychiatric comorbidity.^13^ Studies by Mohr et al.^14^ and Corbisiero et al.^15^ indicated high rates of comorbidity of ADHD with other psychiatric conditions. Of notable consideration is the high rate of comorbidity between ADHD and substance use disorders (SUD), with literature indicating that risk of development of one or more SUDs in adult ADHD is twice as high as that seen for the general population.^16^ Additionally, the presence of ADHD has been reported to modulate the course of SUDs, with its presence being associated with earlier age of onset, more rapid induction of SUD, more severe presentation of SUD and poorer remission rates.^16^ However, despite this known association, distinguishing ADHD as a co-occurring disorder in SUD subjects presents its own challenges given that each condition can impact the other’s presentation, prognosis and severity.^17^

These examples of adult ADHD recognition challenges are emphasised when considering that characteristics often accompanying the disorder were not explicitly associated with ADHD diagnostic criteria prior to DSM-5 publication.^18^ Furthermore, symptoms may vary among patients, evolve over time and mimic symptoms of other disorders.^19,20^ Additional challenges in ADHD research which predated DSM-5 were as follows:

1. the criterion that clinically significant ADHD symptomology should have been present from childhood, prior to the age of 7 years
2. problems with retrospective corroboration of symptoms in adults.^21,22^

The net result of these diagnostic limitations was to make the diagnosis of ADHD in adults contentious. Therefore, the disorder is often under-diagnosed and under-recognised in adults, emphasising the need for a valid screening instrument.

## Diagnostic tools and scales for attention-deficit/hyperactivity disorder

The DSM-5^1^ (together with its earlier editions) and the International Classification of Diseases (ICD-10)^23^ were the diagnostic manuals available for the diagnosis of ADHD at the time the study was conducted.^3^ It is acknowledged that ICD-11 has since been released. For older adolescents and adults, the DSM-5 requires at least five symptoms of hyperactivity-impulsiveness, inattention or both. It also calls for strong evidence that the symptoms interfere with, or reduce the quality of social, academic or occupational functioning. The predominantly hyperactive-impulsive, predominantly inattentive and combined subtypes are maintained, but are now called presentations, because their poor temporal stability has resulted in their validity being questioned.^1,24,25^ To facilitate ease of application of these diagnostic criteria, a number of screening tools based on their listed symptom categories and descriptions have been developed. These include Conners Comprehensive Behaviour Rating Scales (CBRS),^26^ Conners Adult ADHD Diagnostic Interview (CAADID),^27^ Mini International Psychiatric Interview (MINI PLUS) ADHD module,^28^ Brown ADD Scale (BADDS) Diagnostic Form, Wender Utah Rating Scale (WURS)^10^ and the World Health Organisation’s (WHO) Adult ADHD Self-Report Scale (ASRS).^29^

Self-report measures are frequently used to confirm ADHD symptoms and levels of impairment in adolescents and adults. There is strong evidence that adults are reliable reporters of current ADHD symptoms and that adults’ self-ratings and informant ratings are highly correlated.^22,30^

To investigate the validity of adult self-report questionnaires with respect to the factor involving corroboration of both retrospectively reported childhood symptoms and current ADHD symptoms, the study by Murphy and Schachar^22^ required adult subjects to complete two questionnaires. Each one related to either childhood or current ADHD symptoms, with a parent and partner completing similar corresponding questionnaires. The subjects were not drawn from an ADHD population, nor were they assessed for ADHD.^22^ When the completed questionnaires were compared and assessed, statistically significant correlations were found.^22^ These results suggest that, when questions hold a degree of specificity, adult self-assessment of ADHD symptoms can be considered accurate.^22^

The Adult ADHD Self-Report Scale Version 1.1 (ASRS v1.1) was developed jointly by the WHO and Kessler et al.^27^ in 2005 and is a reliable and valid self-administered screener for evaluating current ADHD symptoms in adolescents and adults.^31,32^ The ASRS v1.1 consists of 18 questions (nine questions probe for inattention and another nine questions probe hyperactivity and/or impulsiveness) based on the criteria used for diagnosing ADHD in the DSM-IV (Text Revision). Six of these items – the ASRS Part A (ASRS-A) – have been found to be most predictive of symptoms consistent with a diagnosis of ADHD.^33,34,35^

The design of the ASRS allows for collection of ADHD symptoms in the context of adulthood by relating symptoms to adult situations such as work, tasks or projects instead of play or school activities.^36^ The investigation by Kessler et al.^29^ reported very good reproducibility of overall clinical evaluations for use of the six-question screener, with the full evaluation enabling refinement of the clinical classification and showing good correlation with clinician-rated symptom severity. The ASRS sensitivity within the survey population used by Kessler and colleagues was determined to be 68.7%, with a specificity of 99.5%, yielding total classification accuracy of 97.9%.^10^ However, it is noted that specificity may be lower in clinical samples with high rates of psychiatric comorbidities, with a substance using sample yielding a higher sensitivity of 87.5% but a lower specificity of 68.6%.^10^ This underpins the need to ensure that proper diagnostic assessment is completed following screening activities.

This screening tool has been translated into many different European and Asian languages and is widely used internationally.^37^ According to Yeh et al.,^36^ the investigation into the use of the Chinese version of the ASRS in Taiwanese adults was thought to be the first study to examine the psychometric properties of this tool outside of the United States. The Chinese version was found to be reliable and valid.^36^ An investigation into the use of the Korean version of the ASRS showed that the tool displayed good reliability and validity.^37^

## Use of the Adult Attention-Deficit/Hyperactivity Disorder Self-Report Scale in Africa

Research into the use of the ASRS in African populations is, however, limited. It has not yet been translated into African languages. Some schools of thought hypothesise that the diagnosis of ADHD is a construct of modern Western culture,^38^ with superficial assessment of the rates of the condition reported for both Africa and the Middle East in the metaregression analysis conducted by Polanczyk et al.^39^ supporting this notion. Although the rates of ADHD reported for these locations were comparatively lower than those for other geographic areas, the authors stated that this finding should be interpreted carefully given that the number of available studies for inclusion in their review was limited in comparison to other geographic areas.^39^ This study did, however, determine that the reported variability in prevalence rates across the world was mainly because of the methodological differences in the analysed studies.^39^ Thus, there is scope for the application of a standardised screening tool within African populations. Such a screening tool should be appropriately translated to facilitate understanding and allow for comparison with international prevalence rates.

The ASRS has been updated for DSM-5 criteria by Ustun et al.,^40^ and the operating characteristics were improved. The new screening scale is short, is easily scored, detects the majority of cases and has a high predictive value.

## Study aim

The primary aim of the study was to investigate the use of the ASRS in a South African setting.

### Objectives

The objectives of the study were as follows:

- to screen for ADHD using the ASRS in a psychiatric patient population in the public health sector
- to administer the ASRS using psychiatrically trained nurses working within the selected environment
- to establish the potential prevalence of ADHD in the study population
- to identify substance use pattern differences between groups defined by the ADHD screening results and the presence of EF and emotional control (EC) deficits

## Methods

### Setting

A patient population in Port Elizabeth, South Africa, was identified representing a developing or low- and middle-income country (LMIC) patient population. This population was used to perform a preliminary investigation into the use and application of the ASRS within a South African context. The setting was an acute psychiatric treatment unit that is utilised for the stabilisation of psychiatric patients presenting with acute mental illness (with patients being either discharged from care or transferred to an acute psychiatric hospital after an appropriate duration of treatment).

Consultation with physicians in the facility indicated that routine screening for psychiatric conditions such as mood disorders and schizophrenia in subjects was established; however, screening for the presence of ADHD was generally not conducted. Given the high rate of comorbidity seen in psychiatric populations and the potential for identification of previously undiagnosed ADHD in adults, the unit was considered to be an appropriate setting for conducting the study.

### Sampling

A convenience sample was used with a target for screening of 30 patients. The sample size was not statistically determined, but was applied because of the study being exploratory in nature with limited resources available. Three psychiatrically trained nurses were utilised as fieldworkers. Selection of the nurses was performed in consultation with the facility physicians. Exclusion criteria were participants who did not complete primary school education and those with a history of head injuries. Training was provided to fieldworkers during April 2016. Each nurse screened 10 patients, with data collection being completed between July and December 2016.

Although the ASRS is designed for self-administration, a decision was taken for the screening tool to be administered to aid understanding of possible language or cultural peculiarities which were thought to provide challenges with translation and interpretation of the type of information required from the participants. The data collection duration was not preset prior to initiation of the study for the following considerations:

- Administration time required for each screening could not be accurately determined based on the training provided to the representative fieldworker.
- Data collection was performed within the facility during the participants’ inpatient admission, thus limiting the time available for identification of suitable participants and administration of the data collection tools.
- Use of private consultation areas was required, the availability of which was limited.
- Fieldworkers had to perform the screening during hours in which they were not on duty.

### Assessment method

The ASRS was used with another data collection tool suitable for use in psychiatric patients, which included details on demographic information and substance use and consumption. The data collection pack consisted of the following sections and summarised detail regarding the particular information gathered within each section:

1. **Demographic information:** Introducing questions including the differential diagnosis, endorsement of the presence or absence of SUDs upon admission as per DSM-IV classification, general demographical considerations, level of education and employment status.
2. **ASRS v1.1 (expanded):** Questions 1 through 18 examine ADHD-associated symptoms as per DSM-IV, questions 19 through 32 examine EF and EC and questions 33 and 34 examine impulsiveness as per DSM-5.
3. **Substance consumption information:** A comprehensive list of various intoxicating substances to examine historical or present (active within the month preceding admission) consumption of substances by patients and duration(s) of long-term substance use where present.
4. **ADHD treatment details (if applicable):** To assess for current or historical use of ADHD-indicated medication.

It was estimated that administration of the data collection pack would require a period of 1 hour per interviewed participant.

### Statistical analysis

Data were analysed using Statistica v. 12 (Dell Software). Analysis of variance (ANOVA) models were employed to determine differences in age, gender, inattentive and hyperactive/impulsive scores, EFs, EC and age when substance consumption started. The results were analysed as a function of ASRS results (ASRS positive vs. ASRS-negative). Fisher’s exact test was used for 2 × 2 contingency tables (gender, previously diagnosed ADHD, previous MPH medication and differences in the substances used). Differences in other categorical data (ethnicity, home language, employment education level and clinical diagnosis) were established by chi-square test.

## Ethical consideration

The research proposal wherein the procedures for collection of data from human participants were described was in accordance with the ethical standards of the Research Ethics Committee (Human) of Nelson Mandela University and with the 1964 Helsinki declaration and its later amendments (H14-HEA-PHA-081). Permission to perform the study at the selected unit was provided by the hospital manager. The study fieldworkers selected candidates for the investigation and informed consent was obtained from participants prior to administration of the data collection tools. The informed consent procedure allowed for patients to endorse if they granted permission for the results of the screening to be shared with their attending health-care team with consideration for potential modification of the respective treatment plan(s); however, follow-up related to this aspect was not undertaken within the study and has not been reported upon.

## Results

### Participant demographic information

A total of 30 questionnaires were completed. All participants were South African, with two-thirds of the participants being men (66.7%, *n* = 30). The ethnicity of participants varied with the highest ethnic representation being black people (46.7%), followed by mixed race people (23.3%) and white people (3.3%). The remaining participants did not clearly endorse ethnicity (26.7%).

Half of the participants indicated isiXhosa as their home language, 36.7% were Afrikaans speaking (11, *n* = 30) and 13.3% were English speaking (4, *n* = 30). More than half the participants were unemployed (55.0%, *n* = 30) and 50.0% did not complete the final year of their secondary education (grade 12).

The diagnoses endorsed upon admission varied; refer to Table 1. Substance use disorders were definitively stated for 16.7% of participants (5, *n* = 30). The admission diagnosis was not stated for 40.0% of participants (12, *n* = 30). In some instances, conditions such as bipolar disorder and substance-induced psychosis were the considered differential diagnoses.

**TABLE 1:** Comparison between attention-deficit/hyperactivity disorder-positive and attention-deficit/hyperactivity disorder-negative screened psychiatric patients.

Variable	ADHD				No ADHD				*p*
	*n*	%	Mean	s.d.	*N*	%	Mean	s.d.	
Age	13	43.3	28.15	10.72	17	56.7	30.00	12.32	0.87
**Gender**									0.74
Male	11	36.7	-	-	9	30.0	-	-	-
Female	2	6.7	-	-	8	26.7	-	-	-
**Ethnicity**									0.05
Black people	7	23.3	-	-	8	26.7	-	-	-
Mixed race	4	13.3	-	-	3	10.0	-	-	-
White people	2	6.7	-	-	0	0.0	-	-	-
Not stated	0	0.0	-	-	6	20.0	-	-	-
**Home language**									0.73
isiXhosa	7	23.3	-	-	8	26.7	-	-	-
Afrikaans	5	16.7	-	-	6	20.0	-	-	-
English	1	3.3	-	-	3	10.0	-	-	-
**Employment**									0.38
Employed	2	6.7	-	-	3	10.0	-	-	-
Unemployed	6	20.0	-	-	10	33.3	-	-	-
Part-time	4	13.3	-	-	2	6.7	-	-	-
Student	1	3.3	-	-	2	6.7	-	-	-
**Education level**									0.13
Primary	1	3.3	-	-	1	3.3	-	-	-
Secondary	9	30.0	-	-	4	13.3	-	-	-
Matric	2	6.7	-	-	8	26.7	-	-	-
Post-matric	1	3.3	-	-	4	13.3	-	-	-
**Clinical diagnosis**									0.82
AIDS dementia	0	0.0	-	-	1	3.3	-	-	-
SUD	3	10.0	-	-	2	6.7	-	-	-
Mood disorders	3	10.0	-	-	3	10.0	-	-	-
Personality disorders	0	0.0	-	-	1	3.3	-	-	-
Psychosis	2	6.7	-	-	3	10.0	-	-	-
Not stated	5	16.7	-	-	7	23.3	-	-	-
**ADHD variables**									
Previously diagnosed	1	3.3	-	-	0	0.0	-	-	0.43
MPH medicated	2	6.7	-	-	0	0.0	-	-	0.09
Inattentive score	13	43.3	24.85	2.70	17	56.7	13.82	6.85	< 0.001**
Hyp/Imp score	13	43.3	20.08	4.66	17	56.7	10.94	5.97	< 0.001**
Executive functions	13	43.3	18.85	7.68	17	56.7	4.00	5.79	< 0.001**
Emotional control	13	43.3	9.23	4.44	17	56.7	2.71	3.35	< 0.001**
**Substance use**									
Age when started	13	43.3	15.23	2.55	15	46.7	18.00	7.34	0.21
Alcohol	7	23.3	-	-	5	16.7	-	-	0.16
Cannabis	10	33.3	-	-	5	16.7	-	-	0.03*
Street drugs	7	23.3	-	-	4	13.3	-	-	0.09
Polysubstance use	10	33.3	-	-	3	10.0	-	-	0.04*

Note: ANOVA was used to establish differences in age, inattentive-, hyperactive/impulsive-, executive function- and emotional control scores and start age of substance use. Substance use information reported per substance type and for polysubstance use only refers patients with a current SUD diagnosis. Fisher’s exact test was used to establish differences in gender, previously diagnosed ADHD, previous MPH medication and drug use. χ^2^ was used to establish differences in ethnicity, home language, employment, education level and clinical diagnosis.

ADHD, attention-deficit/hyperactivity disorder; SUD, substance use disorder; MPH, methylphenidate, Hyp/Imp, hyperactive/impulsive; s.d., standard deviation.

* *p* < 0.05,

** *p* < 0.001.

### Attention-Deficit/Hyperactivity Disorder Self-Report Scale screening

Challenges with administration of the ASRS were reported by the fieldworkers because of difficulty in interpretation of concepts therein described in the English language. As a result, the estimated interview duration of 1 h was found to be conservative, and the data collection process was more time consuming than initially anticipated.

Cronbach’s α was calculated for both the ASRS-A (first six questions) and the full scale ASRS (18 questions) for the present sample. Cronbach’s α for the ASRS-A was 0.72, which is an acceptable value, while for the ASRS-18 it was 0.93, which is considered excellent. The internal consistency, which is a measurement of scale reliability, is adequate for the ASRS-A and excellent for the full-scale ASRS for the sample under investigation.

Table 1 provides the results of ADHD screening. For ease of reporting, the group of participants in which the ASRS screening indicated the possibility of an ADHD diagnosis will be referred to as ‘ASRS-positive’, with the converse of ‘ASRS-negative’ used to describe the remaining participants in relation to screening results. With the completion of ASRS-A, 33.3% of participants screened positively for ADHD (10, *n* = 30). This increased to 43.3% upon completion of the 18-question screening tool (ASRS-18) (*n* = 13). It is noted that 6.7% of participants indicated current or previous pharmacotherapy with methylphenidate, possibly signifying previous ADHD diagnosis (a confirmed diagnosis was only indicated for one participant, and the admission differential diagnosis was not available for either participant). The predominantly inattentive presentation was most commonly identified within the ASRS-positive group (76.9%, *n* = 10).

Analysis of ADHD symptom score differences between genders revealed that inattention symptoms were significantly more severe in male participants, with the mean score for inattention being 21.00 ± 6.29 out of a possible 36 (*n* = 20), compared to 13.80 ± 8.40 for females (*n* = 10) (*p* = 0.013). Hyperactive/impulsive symptoms were also more severe for male participants, but the difference between genders was not significant (*p* = 0.101). The ASRS-positive group showed significantly increased severity of hyperactive/impulsive and inattention symptoms of ADHD, as well as problems with EF and EC than the ASRS-negative group.

### Reported substance use and consumption

Considerations for the presence of SUDs in participants were based on the stated diagnosis (where this was available); endorsement of one or more SUDs being present by the indication of alcohol, cannabis and/or drug abuse/dependence (as per DSM-IV); and assessment of the reported substance consumption obtained with completion of the data collection pack. Previous or ongoing substance consumption was reported by 93.3% of participants (28, *n* = 30), whereas diagnosis of a current SUD was endorsed for 73.3% of participants (22, *n* = 30). Thus, 92.3% of the ASRS-positive group had one or more SUDs (12, *n* = 13), while 58.8% of the ASRS-negative group were identified as having SUDs (10, *n* = 17). Substances were divided into alcohol, cannabis and street drug use (cocaine and methamphetamine) and comparisons were made between the ASRS-positive and ASRS-negative groups for patients with a current SUD. The number of ASRS-positive participants endorsed for substance use of each substance division was greater than the representation for ASRS-negative participants; however, no significant differences were found for use of alcohol and street drugs. Significant differences were identified for cannabis and polysubstance use (*p* = 0.03 and *p* = 0.04, respectively).

When comparing the age of initiation of substance consumption for the study population, significant differences were not found between the two groups. The ASRS-positive participants however were generally younger, with an average age of 15.23 ± 2.55 against 18.03 ± 7.34 years.

## Discussion

This study showed that there is merit in investigating the possibility of underlying ADHD in psychiatric populations. Using the endorsement of either current or historical methylphenidate use, the presumed initial rate of ADHD in the study population was 6.7%, which is in line with the estimated prevalence rate of 1.0% – 7.3% in adults^11^ and the global prevalence rate of 5.3% in children and adolescents.^39^ The results of screening with ASRS-A indicated a prevalence of 33.3% (10, *n* = 30) in our study sample, with an increase to 43.3% (*n* = 13) with completion of the full ASRS. Both participants with a history of methylphenidate therapy were within the ASRS-positive group. Although the ADHD diagnosis was not verified by a suitably qualified physician for the purpose of this investigation, these rates are reflective of the assertion that historical diagnostic shortcomings and an array of other potential diagnoses could impair identification of ADHD. The need for further exploration of ADHD being an undetected condition in existing psychiatric adult populations is emphasised by the estimation that most adults with ADHD will experience one or more co-morbid psychiatric disorders during their lifetime and that high comorbidity rates are a distinct feature in adults and children with ADHD.^41^

### Attention-Deficit/Hyperactivity Disorder in African populations

Considering participant ethnicity in relation to ASRS-positive participants, 6 out of the 10 ASRS-A participants were black people, 3 were mixed race people and one was unknown. Following the completion of the 18-question screener, the demographics were 7 black people, 4 mixed race people, one white person and one unknown. Thus, more than half of the participants in whom a diagnosis of ADHD was suspected were black people, which concurs with literature reports of inter-racial variance in ADHD prevalence rates being more dependent on the application of suitable screening tools than any distinct cultural differences.^9,39^ This finding supports the need for appropriate translation of the ASRS to better facilitate investigation of this disorder in African populations.

### Executive function, emotional control and attention-deficit/hyperactivity disorder presentations and substance use trends

The gender distribution among the ASRS-positive group was consistent with the existing literature of higher ADHD prevalence rates in males compared to females. It has been noted, however, that variability in the prevalence between the genders tends to reduce with age as more women become diagnosed in adulthood because of the impairment arising from the internalised symptoms which may have been overlooked in childhood.^10^ The identified presentations of the disorder were skewed towards the inattentive subtype, which is consistent with the study by Cumyn et al.^41^ wherein this was the predominant presentation; however, the literature suggests that the majority of cases are the combined presentation.^42^ The combined presentation is thought to affect both genders equally, whereas the inattentive presentation is more common in women.^42^ The predominantly hyperactive/impulsive subtype is considered the rarest presentation.^42^

The significant differences identified between the ASRS-positive and ASRS-negative groups for EF and EC are consistent with literature stating that such deficits are strongly associated with ADHD.^43,44^ The executive control and cortico-cerebellar networks coordinate executive functioning, which can be described as planning, goal-directed behaviour, inhibition, working memory and the flexible adaptation to context. These networks are underactivated and have lower internal functional connectivity in individuals with ADHD compared with individuals without the disorder.^43^ Persons with ADHD show impairments in judgement, organisation, planning and decision-making as well as in behavioural disinhibition and cognitive flexibility.^4,45,46^ EC deficits include irritability, hot temper, low frustration tolerance, impatience and sudden unpredictable shifts towards negative emotions such as anger, dysphoria and sadness, occurring in an intensity that is considered inappropriate in relation to the situational context, age and developmental stage.^47,48^ It predicts poorer social outcome and peer rejection.^49^ With reference to Barkley’s model,^4,50^ impairments in EF and EC can be explained by a core inhibition deficit; insufficient emotional regulation is considered to represent one consequence of deficient executive inhibitory control. The consequences of impaired response inhibition associated with ADHD to emotional competence include increased emotional reactivity, decreased frustration tolerance and diminished ability to self-regulate emotions. Both motivational and executive processes are likely involved in emotional responding.^48^

It was reported that trends identified for cannabis- and polysubstance use were significantly different between the ASRS-positive and negative groups. These findings were consistent with the results reported in the study by Fabricius et al.^51^ It is notable that these consistencies were identified between studies conducted within South Africa for both cannabis- and polysubstance use, with specific consideration given to the association with cannabis given that this would appear to contrast against the assumption that ADHD would have a greater association with stimulant abuse.^51^ It was considered by Fabricius et al.^51^ that this may be related to easy accessibility of cannabis in South Africa. The association with polysubstance use could be interpreted as being consistent with the literature stating that ADHD is associated with a more severe presentation of SUD.^16^

### Study strengths and limitations

This study provided evidence within an African setting which concurs with existing literature regarding the association between mental illness, ADHD and substance use. Given the previously stated lack of data on ADHD from Africa, this investigation contributes to the greater body of literature specifically pertaining to ADHD, but also its relation to substance use and co-morbid psychiatric conditions. The determination of Cronbach’s α in such a setting is valuable for guiding the use of the ASRS within similar study populations and thus aiding further efforts towards gathering the much needed data on this condition in Africa. These study strengths are, however, impacted by a number of limitations which are discussed below:

1. The sample size was small, and thus, the generalisability and reliability of the study are limited and would require reproduction on a larger scale in order to appropriately identify trends.
2. A convenience sample was used, and thus, a selection bias existed in the study population. These results cannot be applied to the general population and can only be considered for potential generalisation in similar clinical settings.
3. Despite investigations that support the validity of self-report questionnaires, recall bias in this specific patient population may have resulted in skewing of the results which impairs the study reliability and generalisability.
4. As the participants were sourced from a clinical setting, the nature and severity of the current illness(es) experienced by the participants may have interfered with the integrity of data collection tools, resulting in comorbidity bias. Additionally, the provisional or differential diagnoses upon admission were not available for all participants, reducing the potential for analysis directed by confirmed diagnostic information.
5. The English version of the ASRS was used for a population with varying home languages. The specific limitation with respect to the sample was the lack of a validated format of the ASRS in Xhosa. Thus, there is the risk of inappropriate interpretation of the data collection tool questions which may have skewed the results in different possible ways, although the Cronbach’s α indicated that the instrument was reliable for the sample.
6. Despite the fieldworkers being psychiatrically trained and thus ideal for interactions with subjects within the study sample, the previously stated limitations relating to the study sample and language barriers were apparent with respect to appropriate interpretation and administration of the ASRS. Such challenges could be mitigated by the presence of a psychometrist; however, the availability of such professionals in developing areas may be limited, and as such, it is imperative that similar investigations in such populations are undertaken by fieldworkers with psychometric assessment experience and/or training. Despite the mentioned obstacles, the statistics done on the responses of the present sample showed that the instrument (ASRS) was suitable for the population under investigation as it showed to be reliable (good internal consistency).

## Recommendations

Despite the stated limitations, this study provides evidence to support a recommendation to include ADHD screening in acute inpatient settings in the South African context. It is noted that generalisability of the study is limited, and, thus, a further recommendation would include continued investigation into use of the ASRS on a larger scale with improved study design and controls, such as ensuring appropriate diagnostic assessment for ASRS-positive participants to allow for a true indication of the prevalence of ADHD in the selected population. However, application of the ASRS on its own may be insufficient. The diversity of culture and language in South Africa creates a need for appropriate translation and validation of this instrument into indigenous South African languages so as to facilitate improved completion and/or administration of the tool across different ethnic groups.

## Conclusion

Although challenges related to language and duration of the assessment, the target number of screenings was completed within a culturally diverse sample of psychiatric patients. Determination of Cronbach’s α for both the ASRS-A and ASRS-18 within the study population revealed the internal consistency to be adequate for the ASRS-A and excellent for the full scale ASRS. The potential prevalence of ADHD within the study population was 33.3% (ASRS-A) and 43.3% (ASRS-18), whereas the presumed initial prevalence estimation was only 6.7% (*n* = 30), supporting the recommendation that ADHD screening be included within the standard assessment in such populations. Substance consumption and use was widespread within the study population, with significant differences being identified between the ASRS-positive and ASRS-negative groups for cannabis- and polysubstance use. A significant increase in severity of executive functioning and EC problems was also associated with the ADHD-positive group which was consistent with the available literature indicating the association between such deficits and ADHD.

## References

[1] American Psychiatric Association Diagnostic and statistical manual of mental disorders. 5th ed Arlington, VA: American Psychiatric Association; 2013.

[2] Hall C, Newell K, Taylor J, Sayal K, Swift K, Hollis C ‘Mind the gap’ – Mapping services for young people with ADHD transitioning from child to adult mental health services. BMC Psychiatry. 2013;13(1):1–8. 10.1186/1471-244X-13-186 23842080 PMC3717001

[3] National clinical practice guideline number 72 (update) Attention deficit hyperactivity disorder: Diagnosis and management of ADHD in children, young people and adults. National Collaborating Centre for Mental Health. London: The British Psychological Society and The Royal College of Psychiatrists; 2013.

[4] Barkley R Behavioral inhibition, sustained attention, and executive functions: Constructing a unifying theory of ADHD. Psychol Bull. 1997;121(1):65–94. 10.1037/0033-2909.121.1.65 9000892

[5] Brown T ADD/ADHD and impaired executive function in clinical practice. Curr Psychiatry Rep. 2008;10(5):407–411. 10.1007/s11920-008-0065-7 18803914

[6] Ciuluvica C, Mitrofan N, Grilli A Aspects of emotion regulation difficulties and cognitive deficit in executive functions related of ADHD symptomatology in children. Procedia Soc Behav Sci. 2013;78:390–394. 10.1016/j.sbspro.2013.04.317

[7] Coghill D, Seth S, Matthews K A comprehensive assessment of memory, delay aversion, timing, inhibition, decision making and variability in attention deficit hyperactivity disorder: Advancing beyond the three-pathway models. Psychol Med. 2014;44(09):1989–2001. 10.1017/S0033291713002547 24176104

[8] Barkley R Why emotional impulsiveness should be a central feature of ADHD. ADHD Rep. 2010;18(4):1–5. 10.1521/adhd.2010.18.4.1

[9] Bolea B, Adamou M, Arif M, et al. ADHD matures: Time for practitioners to do the same? J Psychopharmacol. 2012;26(6):766–770. 10.1177/0269881111410898 21890596

[10] Kooij SJJ, Bejerot S, Blackwell A, et al. European consensus statement on diagnosis and treatment of adult ADHD: The European Network Adult ADHD. BMC Psychiatry. 2010;10(64):1–24. 10.1186/1471-244X-10-67 20815868 PMC2942810

[11] Schmidt S, Petermann F Developmental psychopathology: Attention deficit hyperactivity disorder (ADHD). BMC Psychiatry. 2009;9(58):1–10. 10.1186/1471-244X-9-58 19761584 PMC2751746

[12] Spencer TJ, Biederman J, Mick E Attention-Deficit/Hyperactivity Disorder: Diagnosis, lifespan, comorbidities and neurobiology. J Pediatr Psychol. 2007;32(6):631–642. 10.1093/jpepsy/jsm005 17556405

[13] Barkley R, Brown T Unrecognized Attention-Deficit/Hyperactivity Disorder in adults presenting with other psychiatric disorders. CNS Spectr. 2008;13(11):977–984. 10.1017/S1092852900014036 19037178

[14] Mohr P, Látalová K, Čeplová Z, et al. Adult ADHD in a general psychiatric population. Eur Neuropsychopharmacol. 2014;24:S350–S351. 10.1016/S0924-977X(14)70558-9

[15] Corbisiero S, Hartmann-Schorro R, Riecher-Rössler A, Stieglitz R Screening for Adult Attention-Deficit/Hyperactivity Disorder in a psychiatric outpatient population with specific focus on sex differences. Front Psychiatry. 2017;8:1–8. 10.3389/fpsyt.2017.00115 28713294 PMC5491936

[16] Sobanski E Psychiatric comorbidity in adults with attention-deficit/hyperactivity disorder (ADHD). Eur Arch Psychiatry Clin Neurosci. 2006;256(1):26–31. 10.1007/s00406-006-1004-4 16977548

[17] Langås A-M, Malt UF, Opjordsmoen S Comorbid mental disorders in substance users from a single catchment area – A clinical study. BMC Psychiatry. 2011;11(25):1–12. 10.1186/1471-244X-11-25 21314980 PMC3042931

[18] Chao C, Gau S, Mao W, Shyu J, Chen Y, Yeh C Relationship of attention-deficit-hyperactivity disorder symptoms, depressive/anxiety symptoms, and life quality in young men. Psychiatry Clin Neurosci. 2008;62(4):421–426. 10.1111/j.1440-1819.2008.01830.x 18778439

[19] Adler L, Spencer T, Faraone S, et al. Validity of pilot adult ADHD Self-Report Scale (ASRS) to rate adult ADHD symptoms. Ann Clin Psychiatry. 2006;18(3):145–148. 10.1080/10401230600801077 16923651

[20] Adler L, Newcorn J Administering and evaluating the results of the Adult ADHD Self-Report Scale (ASRS) in adolescents. J Clin Psychiatry. 2011;72(06):e20 10.4088/JCP.10081tx2c 21733473

[21] American Psychiatric Association Diagnostic and statistical manual of mental disorders [Text revision (DSM-IV-TR]). 4th ed Washington, DC: American Psychiatric Association; 2000.

[22] Murphy P, Schachar R Use of self-ratings in the assessment of symptoms of Attention Deficit Hyperactivity Disorder in adults. Am J Psychiatry. 2000;157(7):1156–1159. 10.1176/appi.ajp.157.7.1156 10873926

[23] World Health Organization International statistical classification of diseases and related health problems 10th revision (ICD-10). Geneva, Switzerland: World Health Organization; 2010.

[24] Levy F DSM-5, ICD-11, RDoC and ADHD diagnosis. Aust N Z J Psychiatry. 2014;48(12):1163–1164. 10.1177/0004867414557527 25366330

[25] Willcutt E, Nigg J, Pennington B, et al. Validity of DSM-IV attention deficit/hyperactivity disorder symptom dimensions and subtypes. J Abnorm Psychol. 2012;121(4):991–1010. 10.1037/a0027347 22612200 PMC3622557

[26] Conners CK Conners’ Comprehensive behavior rating scales. Toronto, ON: Multi-Health Systems; 2008.

[27] Epstein J, Johnson DE, Conners CK Conners’ Adult ADHD Diagnostic Interview for DSM-IV. North Tonawanda, NY: Multi-Health Systems; 2008.

[28] Sheehan DV, Lecrubier Y, Sheehan KH, et al. The Mini-International Neuropsychiatric Interview (MINI): The development and validation of a structured diagnostic psychiatric interview for DSM-IV and ICD-10. J Clin Psychiatry. 1998;59 Suppl 20:22–33. 9881538

[29] Kessler RC, Adler L, Ames M, et al. The World Health Organization adult ADHD self-report scale (ASRS): A short screening scale for use in the general population. Psychol Med. 2005;35(2):245–256. 10.1017/S0033291704002892 15841682

[30] Downey KK, Stelson FW, Pomerleau OF, Giordani B Adult attention deficit hyperactivity disorder: Psychological test profiles in a clinical population. J Nerv Ment Dis. 1997;185(1):32–38. 10.1097/00005053-199701000-00006 9040531

[31] Adler LA, Shaw DM, Spencer TJ, et al. Preliminary examination of the reliability and concurrent validity of the Attention-Deficit/Hyperactivity Disorder Self-Report Scale v.1.1 symptom checklist to rate symptoms of Attention-Deficit/Hyperactivity Disorder in Adolescents. J Child Adolesc Psychopharmacol. 2012;22(3):238–244. 10.1089/cap.2011.0062 22537184

[32] Hines JL, King TS, Curry WJ The adult ADHD self-report scale for screening for adult attention-deficit-hyperactivity disorder (ADHD). J Am Board Fam Med. 2012;25(6):847–853. 10.3122/jabfm.2012.06.120065 23136325

[33] Gray S, Woltering S, Mawjee K, Tannock R The adult ADHD Self-Report Scale (ASRS): Utility in college students with attention-deficit/hyperactivity disorder. PeerJ. 2014;2:1–17. 10.7717/peerj.324 PMC3970798 24711973

[34] Hesse M The ASRS-6 has two latent factors. J Atten Disord. 2012;17(3):203–207. 10.1177/1087054711430330 22262467

[35] Van de Glind G, Van de Brink W, Koeter M, et al. Validity of the Adult ADHD Self-Report Scale (ASRS) as a screener for adult ADHD in treatment seeking substance use disorder patients. Drug Alcohol Depend. 2013;132(3):587–596. 10.1016/j.drugalcdep.2013.04.010 23660242 PMC4083506

[36] Yeh C-B, Gau SS-F, Kessler RC, Wu Y-Y Psychometric properties of the Chinese version of the adult ADHD Self-report Scale. Int J Methods Psychiatr Res. 2008;17(1):45–54. 10.1002/mpr.241 18286465 PMC6878254

[37] Kim J-H, Lee E-H, Joung Y-S The WHO Adult ADHD Self-Report Scale: Reliability and validity of the Korean version. Psychiatry Investig. 2013;10(1):41–46. 10.4306/pi.2013.10.1.41 PMC3590429 23482673

[38] Timimi S, Taylor E ADHD is best understood as a cultural construct. Br J Psychiatry 2004;184(1):8–9. 10.1192/bjp.184.1.8 14702221

[39] Polanczyk G, De Lima MS, Horta BS, et al. The worldwide prevalence of ADHD: A systematic review and metaregression analysis. Am J Psychiatry. 2007;164(6):942–948. 10.1176/ajp.2007.164.6.942 17541055

[40] Ustun B, Adler LA, Rudin C, et al. The World Health Organization Adult Attention-Deficit/Hyperactivity Disorder Self-Report Screening Scale for DSM-5. JAMA Psychiatry. 2017;74(5):520–526. 10.1001/jamapsychiatry.2017.0298 28384801 PMC5470397

[41] Cumyn L, French L, Hechtman L Comorbidity in adults with Attention-Deficit Hyperactivity Disorder. Can J Psychiatry. 2009;54(10):673–683. 10.1177/070674370905401004 19835674

[42] Groß-Lesch S, Dempfle A, Reichert S, et al. Sex- and subtype-related differences in the domorbidity of Adult ADHDs. J Atten Disord. 2016;20(10):855–866. 10.1177/1087054713510353 24196345

[43] Faraone SV, Asherson P, Banaschewski T, et al. Attention-deficit/hyperactivity disorder. Nat Rev Dis Primers. 2015;1:1–23. 10.1038/nrdp.2015.20 27189265

[44] Skirrow C, McLoughlin G, Kuntsi J, Asherson P Behavioral, neurocognitive and treatment overlap between attention-deficit/hyperactivity disorder and mood instability. Expert Rev Neurother. 2009;9(4):489–503. 10.1586/ern.09.2 19344301

[45] Arnsten AF, Li BM Neurobiology of executive functions: Catecholamine influences on prefrontal cortical functions. Biol Psychiatry. 2005;57(11):1377–1384. 10.1016/j.biopsych.2004.08.019 15950011

[46] Nigg JT, Blaskey LG, Huang-Pollock CL, Rappley MD Neuropsychological executive functions and DSM-IV ADHD subtypes. J Am Acad Child Adolesc Psychiatry. 2002;41(1):59–66. 10.1097/00004583-200201000-00012 11800208

[47] Asherson P, Chen W, Craddock B, Taylor E Adult attention-deficit/hyperactivity disorder: Recognition and treatment in general adult psychiatry. Br J Psychiatry. 2007;190:4–5. 10.1192/bjp.bp.106.026484 17197649

[48] Sobanski E, Banaschewski T, Asherson P, et al Emotional lability in children and adolescents with attention deficit/hyperactivity disorder (ADHD): Clinical correlates and familial prevalence. J. Child Psychol Psychiatry. 2010;51(8):915–923. 10.1111/j.1469-7610.2010.02217.x 20132417

[49] Maedgen J, Carlson C Social functioning and emotional regulation in the attention deficit hyperactivity disorder subtypes. J Clin Child Psychol. 2000;29(1):30–42. 10.1207/S15374424jccp2901_4 10693030

[50] Barkley RA The executive functions and self-regulation: An evolutionary neuropsychological perspective. Neuropsychol Rev. 2001;11(1):1–29. 10.1023/A:1009085417776 11392560

[51] Fabricius V, Langa M, Wilson K An exploratory investigation of co-occurring substance-related and psychiatric disorders. J Subst Use. 2008;13(2):99–114. 10.1080/14659890701680877

